# Effects of Sport Education Model and Physical Fitness on Motivation and Prosociality for a Healthy Approach in Secondary Students Using an Experimental Design

**DOI:** 10.3390/sports13080274

**Published:** 2025-08-18

**Authors:** Augusto Hoyo-Guillot, Francisco Tomás González Fernández, Pedro Jesús Ruíz-Montero

**Affiliations:** 1 Department of Physiology, Faculty of Education and Sport Sciences, University of Granada, Campus of Melilla, 52005 Melilla, Spain; 2 TEPAS Research Group (HUM 1080), Department of Physical Education and Sports, Faculty of Sport Sciences, University of Granada, 18011 Granada, Spain; 3 Department of Physical Education and Sport, Faculty of Education and Sport Sciences, University of Granada, Campus of Melilla, 52005 Melilla, Spain; 4 Department of Physical Education and Sports, Faculty of Sport Sciences, University of Granada, 18011 Granada, Spain

**Keywords:** sport education model, physical fitness and health, motivation, prosociality, experimental design

## Abstract

Purpose: This study aims to demonstrate the value of physical education (PE) in fostering prosociality and academic motivation through active approaches derived from the sports education model (SEM), in contrast with the traditional methodology (TM). One of the new features introduced is based on an approach focused on physical fitness and health that goes beyond sports practice. Method: A 10-week intervention was conducted with a sample of 127 students (*n* = 127). In total, 63 students (*n* = 63) received an SEM-based intervention and 64 (*n* = 64) received traditional teaching. The effect of these methodologies on motivation, prosocial climate, and the perceived importance of PE has been verified, based on a fitness-oriented proposal. Results: The findings indicate improvements in the SEM group’s prosociality (giving, *p* = 0.015; verbal comfort, *p* = 0.019; solidarity, *p* = 0.039). Additionally, the TM group showed increased importance attributable to PE, though a deterioration in prosocial values was also observed. No evidence was found regarding changes in motivation. Discussion/Conclusion: The implementation of SEM has positive effects on students’ prosociality, whereas the opposite effect can be observed when employing TM. The non-inclusion of sports activities can affect the motivation variable

## 1. Introduction

In today’s rapidly evolving society, the capacity to adapt has become a crucial competency, particularly within educational settings. Educators must address diverse student needs and prepare them for life’s complexities. Modern educational policies underscore the importance of personal and social development as fundamental objectives, especially within physical education (PE) programs [1]. PE is uniquely positioned to cultivate positive attitudes and values that contribute to students’ holistic development, extending beyond motor skill acquisition to encompass vital life competencies such as teamwork, communication, and leadership [2]. However, mere participation in PE classes does not ensure the development of these skills; it necessitates an empowering educational approach that fosters collaboration, self-direction, and effective communication [3]. 

### 1.1. Pedagogical Models vs. Traditional Methodologies

Traditional methodologies (TMs) have historically dominated PE instruction, often emphasizing skill acquisition within a rigid, teacher-centered framework. This approach can inadvertently hinder students’ autonomy and critical thinking abilities [4]. In contrast to traditional teaching methods, modern educational frameworks like the sports education model (SEM) emphasize a more student-centered approach. These models focus on encouraging active participation and fostering personal responsibility among students. Grounded in constructivist and social learning theories, they suggest that students learn best when they are actively involved in their education and can apply what they learn to real-life situations [5]. By moving away from merely delivering content, these approaches prioritize the development of skills that can be used in various contexts. They create opportunities for students to work together, take on different roles within groups, and make decisions that impact on their learning experiences [6]. This shift not only boosts student engagement but also equips them with the tools they need to navigate challenges outside the classroom, preparing them to succeed in a rapidly changing world.

### 1.2. Sport Education

The SEM has gained considerable recognition for its ability to boost student engagement and instill a sense of responsibility in PE settings. By organizing lessons into structured seasons of play, this model allows students to take on various roles such as coach, referee, and player. This diverse participation both enhances their understanding of games and helps in developing important leadership skills [7], promoting dialogue between equals and a progressive autonomy that makes them aware of their own learning, characteristic of dialogic learning [8].

This approach fosters a sense of community and teamwork while also nurturing critical life skills that extend beyond physical sports activity. Research has indicated that students involved in the SEM show significantly higher levels of engagement, motivation, and personal accountability than those in traditional PE settings [9]. This difference has prompted important discussions about how various teaching methods can influence prosocial behavior and academic motivation among secondary students in PE. Incorporating prosocial behaviors into PE curricula is crucial for developing students’ social skills and emotional intelligence. Participating in physical activities that require cooperation, communication, and conflict resolution not only improves students’ abilities to manage social situations but also fosters empathy and respect for others [10]. These skills are essential for building meaningful relationships and creating a positive school environment. However, adding PE to the curriculum is not enough to ensure these benefits. It is vital to cultivate intentional learning environments that actively encourage participation, collaboration, and a sense of belonging among students [11].

Similarly, the SEM is particularly effective in meeting the need for fostering teamwork and peer support, as it naturally creates opportunities for students to engage in prosocial behaviors in real-time [12]. By cultivating a community-focused environment, PE can become a powerful avenue for not only enhancing physical abilities but also for developing crucial life skills that contribute to students’ overall well-being and success in various social situations [13]. Prioritizing prosocial development within PE contexts can lead to more inclusive and supportive educational experiences, highlighting the importance of effective teaching methods such as the SEM in achieving these positive results. In addition to fostering prosocial behaviors, motivation plays a key role in understanding and enhancing student attitudes in PE settings. The motivation of students in PE classes might show several aspects of academic involvement, including intrinsic and extrinsic motivation, as well as amotivation. Using academic motivation, educators can gain valuable insights into students’ motivational profiles, which are essential for their engagement and persistence in PE activities [14]. Research has shown that students with higher intrinsic motivation are more likely to actively participate, set personal goals, and show resilience when facing challenges [15].

### 1.3. The Present Study

This research examined the impact of the SEM intervention on students’ perceptions of PE’s value, their academic motivation, and the prosocial dynamics within the classroom. With these considerations in mind, this study aimed to compare the effects of TM and the SEM approach on students’ perceptions of the importance of PE. Specifically, this research explores how each instructional method affects students’ engagement and the value they place on physical fitness and health. Additionally, we evaluate changes in prosocial behavior among secondary students after implementing either the TM or SEM approaches, focusing on how each model encourages collaboration, teamwork, and the development of social skills within the PE context. Finally, we cannot forget the importance of the variations in academic motivation levels among secondary students resulting from their participation in either the TM or SEM. It is necessary to understand how these different approaches influence students’ overall motivation toward their academic pursuits and learning experiences. By investigating these aspects, this study seeks to offer valuable insights into the broader implications of pedagogical strategies in PE.

## 2. Materials and Methods

### 2.1. Participants

**Figure 1:**
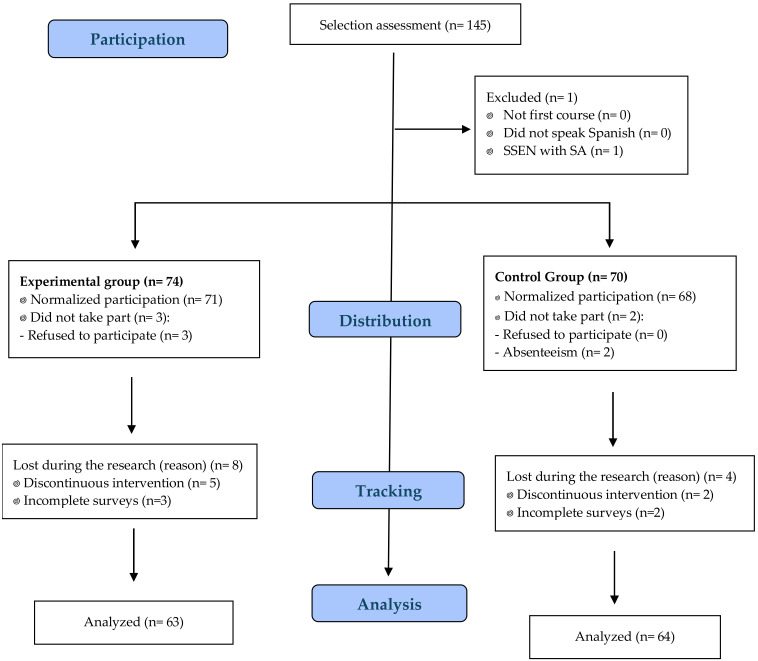
Flow diagram of participants in CONSORT. SSEN: Students with Special Education Needs; SA: Significant Adaptation.

The sample consisted of 127 students (control group—TM = 64; experimental group—SEM = 63) aged between 11 and 14 years (12.8± 0.52). The control group received classes using TM, through teaching styles such as task assignment and microteaching. Conversely, the intervention for the experimental group was characterized by a methodology based on Siedentop’s SEM (6, 7). The selection of classes for the formation of both groups followed a randomized criterion, with the following exclusion criteria: (i) not belonging to the first year of Spanish Secondary Education, (ii) not speaking or having significant difficulties in handling Spanish, and (iii) students with significant curricular adaptations in the subject due to being Students with Special Educational Needs. This entire process can be seen in Figure 1.

This research took place in a public high school in the Autonomous City of Melilla, a Spanish city in North Africa. The parents of the students were required to sign informed consent because the sample was under eighteen years old [16]. After this, the students completed the different surveys with tablets borrowed from the technology department. The surveys collected information on the different variables under study, and other questionnaires collected the demographic data of the sample. 

In Session 0, students completed questionnaires on Google Forms, which stored the collected data. The intervention spanned 16 sessions over 10 weeks during the first term of the 2023/24 school year, specifically, from 2 October to 11 December. Each session lasted 55 min, with a regular frequency of two sessions per week.

The contents introduced during this research were related to fitness and health. These contents were related to didactic programming in the second programming unit, “Get in Shape,” which covers basic knowledge related to physical fitness, such as basic physical abilities and how to train them. The third unit, “Personal Trainers,” focused on different disciplines related to health-related physical fitness, like functional training, CrossFit, and core strength. Finally, the fourth unit, “Healthy Rhythm,” aimed to develop jump rope choreography. The first unit of the course, which was not part of the study but could have influenced its subsequent development, was related to cooperative challenges.

This proposal diverges from traditional approaches related to the sports education model, which typically suggest team-based physical activities to integrate various elements of the SEM [17]. Instead, it incorporates other content areas to promote healthy lifestyles, such as body expression, physical fitness, and small-sided games [18]. We aim to verify that the benefits of this pedagogical model can be applied to other educational proposals. 

Table 1 shows the different elements or characteristics of the SEM established by Daryl Siedentop [6] related to the different units described above, showing how they are included in the pedagogic intervention. Interventions using traditional methodologies have been based primarily on the teacher leading the session; transmitting each of the exercises and execution models directly or through task lists; and addressing the group en masse or in small, non-predetermined groups of four to six members.

### 2.2. Measures

Different research instruments were established, primarily consisting of standardized, specific questionnaires, which are presented below:

- The Importance of Physical Education Questionnaire [19] consists of three items designed to measure adolescents’ perceptions of the relevance and usefulness of PE classes. The items are as follows: (1) I consider it important to receive PE classes, (2) compared with other subjects, I believe PE is one of the most important, and (3) I believe that what I learn in PE will be useful in my life. Each item is rated on a 4-point Likert scale, from 1, “Strongly disagree”, to 4, “Strongly agree.” A factor analysis of these items shows solid internal consistency, with values of 0.827, 0.814, and 0.818 for items 1, 2, and 3, respectively. Additionally, principal component analysis with varimax rotation provides a value of 2.01 for the grouping of factors related to the importance of PE, explaining 67.15% of the total variance. The reliability coefficient is 0.75, indicating a high correlation.
- The School Prosocial Climate Questionnaire [20] consists of 10 items corresponding to 10 predefined categories of prosocial behavior, such as physical help and service, giving or leaving objects, verbal help and comfort, positive evaluation of others (PEO), deep listening, empathy, solidarity, positive presence, and unity. Responses are provided using a 5-point Likert scale (1 = very rarely, 2 = sometimes, 3 = several times, 4 = often, and 5 = almost always). To facilitate data collection, the questionnaire has been adapted for self-assessment to measure how frequently students have experienced these behaviors, rather than co-evaluation. This questionnaire demonstrates high reliability, with values of α = 0.85 in the pre-test phase and α = 0.84 in the post-test phase.
- The Educational Motivation Scale [21] is a questionnaire that has been validated and adapted to Spanish for secondary school students. It evaluates dimensions distributed across seven subscales, including variables such as motivation, external regulation, introjected and identified regulation, and intrinsic motivation (IM) related to knowledge, accomplishment, and stimulating experiences. Each subscale contains 4 items where students explain their reasons for attending school, totaling 28 items. Responses are rated on a seven-point Likert scale, where 1 indicates “not at all,” and 7 indicates “completely,” with a midpoint of 4 indicating “moderately.” This scale demonstrates satisfactory internal consistency, with an average Cronbach’s alpha coefficient of 0.80 and high temporal stability indices, with an average test–retest correlation of 0.75.

### 2.3. Design and Procedure

This study was approved by the Ethics Committee of the University of Granada (No. 3636/CEIH/2023) and is part of the research project “Influence of Dialogic Programs on Motivation, Prosocial Climate, and the Importance of PE in Secondary Education Students.” Additionally, since the intervention takes place in the educational field, approval for its implementation was also obtained from the Ministry of Education and Vocational Training (MEFP).

The Provincial Delegation of Education of Melilla, where this study was conducted, approved the use of questionnaires and the intervention with the participating students. Permission was also obtained from the school administration to conduct the research, as well as consent from adult students and the parents of minor participants. Students completed the questionnaires individually using tablets provided by the school through the Information and Communication Technologies Department. These questionnaires were completed anonymously using an identification code provided by the teachers to monitor completion, both during the pre-test (7–10 days before the intervention) and the post-test (7–10 days after the intervention).

All of this took place during PE class, in a playful session where students attended in groups of eight to complete the questionnaires; this took approximately 15 min. The process was supervised by the teacher in charge of the research, who established student/teacher roles to manage the class during physical activities. The teachers informed the students that participation was voluntary and would not affect their grades. There were no problems in completing the questionnaires.

Table 2 shows the different sessions (S) developed according to the programming of the Physical Education Department for the first year of the General Certificate of Secondary Education. Note that the different units (U) included in this research are part of a larger project called “Oh my coach,” which aims to create personal trainers who promote physical conditions related to health. Furthermore, this session programming is related to distinct phases established by Siedentop et al. [6], evolving from the first phase of directive practice (P1) to a second autonomous phase (P2) to reach a competition phase (P3) and, finally, a final event (P4).

### 2.4. Statistical Analysis

The sociodemographic variables are described as the mean ± standard deviation or the number of high school students (%) for quantitative (age) and categorical variables (gender, religion, and repeating the academic year). Assumptions related to model fit between the groups were assessed, including the independent-sample Student’s *t*-test (normal distribution, homoscedasticity) and the chi-square test for categorical variables. The p-values of post- and pre-differences for both groups of participants (TM and SEM) were obtained using Student’s t-test on items in the PE Importance Scale, the dimensions of the Prosocial Improvement Scale, and the Academic Motivation Scale variables. The magnitude of the differences between the evaluation moments (post and pre) was calculated using the effect size (*η*2), as defined by [22]. According to the same author, the effect size expresses Cohen’s *d* value, interpreted as small (0.2 < *d* < 0.5), medium (0.5 < *d* < 0.8), or large (0.5 < *d* < 0.8).

Finally, we included the changes (post–pre) in the PE Importance Scale, the dimensions of the Prosocial Improvement Scale, and the Academic Motivation Scale variables as dependent variables in separate models. We also included the groups (TM and SEM) as an independent variable. A linear regression of the two models was tested: Model I was unadjusted. Model II was additionally adjusted for gender since gender type is associated with lower PE importance and academic motivation in female high school students [23]. The statistical analysis was conducted with the Statistical Package for Social Sciences (IBM SPSS Statistics for Windows, Version 25.0; IBM, Armonk, NY, USA). The statistical significance was set at *p* = 0.05.

## 3. Results

### 3.1. Sociodemographic Variables

Table 3 shows a descriptive and comparative analysis of the sociodemographic variables of the high students in the TM and SEM groups (age, gender, religion, and whether they have repeated the academic year). Significance is only shown in the age comparison between the two participant groups (*p* < 0.001).

### 3.2. Main Results

The results of the 10-week intervention (Table 4) demonstrated that three dimensions of Prosocial Improvement Scale (giving, verbal comfort, and solidarity) showed post–pre-differences in the SEM group (*p* < *0*.05). However, item 3 of the PE Importance Scale, the PEO category of the Prosocial Improvement Scale, and the variable IM-to know showed differences after the 10-week intervention (*p* < *0*.05).

Table 5 shows the per-protocol analyses of PE importance, prosocial improvement, and academic motivation changes between post- and pre-interventions for the TM and SEM groups. After adjusting for gender (Model II), item 3 of the PE Importance Scale increased by 0.09% in the SEM group, whereas it decreased by 0.4% in the TM group (−0.415, 0.057; *p* = 0.027). Furthermore, the verbal help category of the Prosocial Improvement Scale increased by 0.3% in the SEM group and by 0.02% in the TM group (−0.206, 0.242; *p* = 0.010).

## 4. Discussion

This study analyzed the influence of a SEM intervention on the importance of PE, educational motivation, and prosocial climate among the participating students. Thus, through a quasi-experimental design, we have tried to demonstrate the level of changes produced once the intervention has finished, in contrast to a traditional methodology, with teacher-centered instructional models (e.g., direct instruction or task assignment) in contrast to a student-centered pedagogical SEM model, which is based on behavioristic assumptions [1].

This study focuses on secondary education, specifically in the first year; few studies have focused on this age group, as most are dedicated to higher-level courses with students of greater maturity [24]. Another important demographic aspect is the significant mix of cultures that exists in the center of this research, something that other studies have defined (for example, Puente-Maxera et al. [25]). Specifically, it suggests that this methodology is a source of opportunities to improve these intercultural processes, with special relevance in a globalized world.

Notably, this study did not follow the approach of research whose main content is related to competitive sports [26]. A different theme related to physical condition and health has been addressed, taking advantage of the literacy, enthusiasm, and competency development associated with this methodology [6] to apply it to content that is usually less motivating. Previous proposals focused on content such as EduCrossfit [17] or High Intensity Interval Training (HIIT) have been taken as references. In these studies, not only were the levels of PC improved, but also the students’ perception of it in relation to their health [27].

The number of sessions undertaken in this study (16) is within the range established by other research [28]; however, it is lower than what other authors have proposed, with the idea of extending this methodology beyond one term with the aim of implementing a full season [29].

Not undertaking a sports activity did affect the development of the subjects’ prosocial climate variable (Table 4). Three items experienced a significant improvement or a moderate effect size in categories such as giving, verbal comfort, and solidarity. Giving objects, food, or possessions to others, losing their ownership or use, is a fundamental dimension within prosociality and is an intrinsic value in sports activity [30]. This also applies to verbal comfort, verbal expressions used to reduce the sadness of distressed individuals. There are numerous references that reinforce these results, relating to the implementation of the SEM with an improvement in affective levels [31]. The last item in this variable is solidarity, physical or verbal behaviors that express voluntary acceptance of sharing the consequences, especially painful, of the unfortunate condition, status, situation, or fortune of other people, groups, or countries [19]. This element is even more important in this study, as it exists in an intercultural context; these findings align with those demonstrated by Wang and Cheng [32], who refer to the improvement of some skills in a contextualized manner that are relevant for students in the development of intercultural skills; this reduces conflicts and improves the social climate in the classroom [33].

The SEM has been shown to have positive effects on motivation [34]. However, this study did not find evidence for this variable, which may be due to the lack of inclusion of a particular sport, a motivating element for students that most related studies define as having positive results [35]. This contrasts with the content of physical fitness, which tends to be less motivating for students [36]. Notably, this study has adapted a methodology rooted in sports to a context focused on physical activity and health. The various strategies inherent to the SEM [6] have been tailored within a team-based discipline to the design of gym-based activities, where students assume specific roles and foster their sense of belonging, aiming to improve their physical fitness levels in relation to health. 

Another relevant finding from the control group results is the worsening of outcomes related to the confirmation and positive evaluation of others within the prosocial climate. This can be attributed to interventions with traditional methodologies, as García-González et al. [37] explain in their study, where the group applying teaching styles (as referred to in our study) is identified with worse prosocial outcomes. This negative influence also impacts on the importance students place on physical education, as the results show a regression in the item “What I learn is relevant to my life,” which can be justified by the demotivation associated with this type of methodology [38]. This result contrasts with the improved intrinsic motivation toward knowledge, which may be due to the practice of novel disciplines such as CrossFit [39].

Finally, Table 5 shows the evolution of the variables adjusted by gender, demonstrating a positive evolution in the SEM group regarding the importance students give to PE; this is relevant because the contents developed are related to an active and healthy lifestyle, with a high degree of autonomy, self-efficacy, and satisfaction [40] but also owing to the implementation of an active methodology, as this positive evolution can also be seen in the prosocial climate variable [41]. 

### 4.1. Practical Applications

As a differentiating element, this study has implemented various aspects of the SEM to enhance content directly related to physical fitness and health. Few studies are related to this aspect of physical education, with most focusing on sports activity [42]. Although the different characteristics of this pedagogical model [43] are easily applied to any pedagogical approach, instead of organizing groups into teams, gyms were established with their own identity, as well as mottoes and colors. Additionally, the season began with a preparatory period, where the session structure’s foundations were laid, along with the most important roles for its development. Subsequently, a competition system based on collective performance was developed through collective scoreboards [44], gamified challenges [45], and a qualitative evaluation in the final unit based on rubrics [46]. Finally, two events were organized to demonstrate relevance and real-world transfer, with participants attending a nearby gym and holding a choreography festival.

Although an effect on motivation could not be described, the results suggest that this approach impacts prosociality, as well as the importance students place on physical education, demonstrating that this type of active methodology is applicable to different educational realities, as evidenced by other initiatives [47].

Below, in Table 6, there is a checklist with the main adaptations to elements of the SEM made in this study [7].

### 4.2. Main Limitations

The main limitations of this study lie in the difficulty of evaluating all the elements introduced in a thorough intervention. First, we could not determine the scope of the formative evaluation. Self-assessment and peer-assessment rubrics were used as the main assessment instruments, although this element was not analyzed within the study, as it is considered fundamental to the SEM [48]. Likewise, due to time constraints, roles within the model were not rotated, nor was attention given to the initial characteristics of the students in assigning them. Lastly, as noted above, a specific sports season was not addressed; instead, the programmed contents within the subject’s didactic programming were applied, adapting the model to them and demonstrating its adaptability. Finally, the study lacks interdisciplinarity, which, according to authors like Estrada and Meléndez [49], would allow us to consider the model’s structures and increase motivation.

## 5. Conclusions

In conclusion, the intervention carried out demonstrated the effectiveness of the considered pedagogical model in promoting civic competence, with significant results in social values related to solidarity, respect, and the ability to share. Likewise, active methodologies have positive effects not only on the prosocial climate of the classroom, but also on the importance that students place on PE. However, they have not been shown to increase students’ motivation beyond the implementation of traditional or participatory teaching styles.

## Figures and Tables

**Table 1:** Elements of the SEM.

Unit	Season	Roles	Affiliation	Statistics	Final Event
Get in shape (3S)	Preseason: Teams use fitness to promote their physical condition (PC).	Initial data collection. Team selection. Physical trainer and material manager.	The participants create their own gym and choose a specific color and slogan.	Students conduct physical fitness and health tests; the results are recorded.	There is no final event in this unit.
Personal trainers (7S)	Formal season: Training for CrossFit, functional training, and core exercises. Competition: Fitness challenges.	Journalists take pictures and write articles. Officials oversee the control of repetitions in competition. The coach designs the training sessions.	Teams must identify with their colors and create a shield for their gym and a pep talk that includes the gym’s slogan.	Officials present results and establish classifications. Scores for each group are published on a blog.	Combined competition in a nearby gym. Exercises: Planks, swings, push-ups, burpees, and chin-ups.
Healthy Rythm (6S)	Formal season: Endurance exercises are incorporated: aerobics, body combat, and jump rope.	The role of the choreographer is incorporated. They must design a small and simple jump rope routine.	The participants incorporate a small piece of choreography into the initial pep talk before the competition.	The assessment has a more qualitative approach, except for jump rope exercises.	The best dances are performed by students from other grades.

**Table 2:** Sessions, contents, strategies, and phases of the SEM intervention.

Unit/ Session	Main Content	SEM Strategies	Phase
U2/S1	Initial assessment. Presentation and warm-up. Physical fitness tests: Seat and reach, 4 × 10, horizontal jump, and Harvard step test.	SE groups are established. Physical trainers (PTs) lead warm-up. Affiliation elements (color and slogan) are proposed.	P1
U2/S2	Training session: cooperative physical challenges with a task list. Cool-down (flexibility). Fitness homework.	Role of coach and officials. The coach leads the session, facilitated by the teacher. PT leads stretches.	P1-2
U2/S3	Autonomous warm-up. Fitness games with strength elements. Special methods to improve endurance. CORE exercises.	For the next session, gym-colored uniforms are required. Slogan presentation. Warm-up self-assessment.	P1–2
U3/S1	Functional training and HIIT are presented. Recommended and contraindicated exercises. Typical circuit session with playful forms.	Functional training exercises are provided in virtual classrooms. Trainers configure preparatory circuit session.	P1
U3/S2	Autonomous functional training session. A worksheet is provided to indicate the chosen exercises. Teacher evaluates the session.	The coach and PT configure a complete functional training session. The material manager puts material in the right order.	P2
U3/S3	The basics of EduCrossFit are presented. Recommended exercises and modalities. Guidelines for designing AMRAP.	CrossFit exercises in a virtual classroom. Trainers can configure their preparatory session with six to eight basic exercises.	P1–2
U3/S4	Autonomous CrossFit session is proposed. A 10-min AMRAP is designed. The routine is evaluated by the teacher.	Journalists take pictures of the different exercises. Ethics documents are signed to respect the competition rules.	P2
U3/S5	CrossFit and functional training competition: Four CrossFit exercises and four functional exercises are chosen.	Officials from each gym monitor the execution of the others. Data collection entry in PE’s blog.	P3
U3/S6	CORE session with a task list provided by the teacher. The second part of the session is meant for trainers to improve group performance.	Trainers must indicate the CORE exercises provided, and the journalist takes pictures of their teammates’ planks for feedback.	P1–2
U3/S7	Final strength event: Combined competition integrating a 10-min AMRAP with CORE challenges.	Moving to a nearby gym. Self-management of the competition. A trophy is awarded to the best gym.	P4
U4/S1	Presentation of rhythmic systems. Micro-session involving aerobics and body combat: basic steps. Group jump rope challenge.	PT monitors heart rate. Journalists take on roles as choreographers. A worksheet to create the choreography is provided.	P1
U4/S2	Jump rope session: Basic jumps and choreography guidelines. Jump rope challenges for speed and endurance.	Trainers work with less skilled jumpers and introduce an improvement plan for home. Publication of challenge results.	P2
U4/S3	Choreography creation: General rehearsal of the first minute. Maximum choreography duration: 2 min.	Choreographers decide which jumps are included in the choreography, their order, and formations.	P2
U4/S4	Choreography development. New day of jump rope challenges for speed and endurance.	Self-evaluation of group performance levels. Publication of challenge results.	P2–3
U4/S5	General rehearsal in the auditorium.	Peer evaluation of choreographies using rubrics.	P2
U4/S6	Final cardio Christmas event: Routine demonstrations and voting.	Final event in the school’s auditorium. Christmas festival.	P4

**Table 3:** Sociodemographic variables according to profile of participant groups.

	SEM Group (n = 63)	TM Group (n = 64)	*p*-Value
Age _Means (Standard Deviation)_	12.48 (0.54)	13.13 (0.49)	0.000
Gender	*N* (%)	*N* (%)	0.472
Female	32 (50.8)	36 (56.2)	
Male	31 (49.2)	27 (42.2)	
Other Option	-	1.6	
Religion	*N* (%)	*N* (%)	0.889
Catholic	24 (38.1)	23 (36)	
Islamic	33 (52.4)	36 (56.2)	
Non-Practicing	6 (9.5)	5 (7.8)	
Repeating the Academic Year	*N* (%)	*N* (%)	0.973
No	55 (87.3)	56 (87.5)	
Yes	8 (12.7)	8 (12.5)	

**Table 4:** Per-protocol analysis shows the post–pre-in on PE importance, prosocial improvement, and academic motivation after the 10-week intervention (TM and SEM) in high school students.

	SEM Group (*n* = 69)			TM Group (*n* = 58)				
	Post	Pre	*p*-Value	Cohen’s *d*	Post	Pre	*p*-Value	Cohen’s *d*
Physical Education Importance								
1. I think it is important to receive PE classes.	3.41 (0.83)	3.33 (1.06)	0.626	0.086	3.04 (1.24)	3.37 (0.88)	0.072	0.306
2. I think that PE is one of the most important.	3.01 (0.71)	2.77 (0.88)	0.118	0.301	2.71 (1.00)	2.85 (0.94)	0.373	0.144
3. I think the things I learn in PE will be useful in my life.	3.19 (0.77)	3.09 (0.89)	0.545	0.120	2.81 (1.13)	3.26 (0.82)	0.016	0.455
Prosocial Improvement								
Physical help	3.15 (1.12)	3.12 (1.05)	0.873	0.027	3.53 (1.34)	3.39 (1.29)	0.465	0.106
Physical service	3.15 (1.01)	3.14 (1.10)	0.918	0.009	3.54 (1.41)	3.68 (1.29)	0.496	0.103
Giving	3.53 (0.81)	3.03 (1.25)	0.015	0.474	3.45 (1.43)	3.40 (1.49)	0.830	0.034
Verbal help	3.58 (0.79)	3.68 (0.66)	0.450	0.137	4.25 (1.09)	4.51 (0.75)	0.107	0.491
Verbal comfort	3.58 (0.81)	3.26 (1.09)	0.019	0.333	3.67 (1.29)	3.95 (1.06)	0.129	0.237
PEO	3.44 (0.94)	3.41 (0.90)	0.837	0.032	3.75 (1.23)	4.09 (0.93)	0.037	0.311
Deep listening	3.65 (0.82)	3.53 (0.83)	0.349	0.145	4.01 (1.18)	3.81 (1.21)	0.300	0.167
Empathy	3.30 (0.90)	2.98 (1.19)	0.051	0.303	3.61 (1.26)	3.34 (1.31)	0.182	0.210
Solidarity	3.36 (0.90)	3.03 (1.10)	0.039	0.328	3.56 (1.25)	3.32 (1.21)	0.156	0.195
Positive presence and unity	3.53 (0.81)	3.47 (0.87)	0.662	0.071	3.78 (1.29)	3.85 (1.24)	0.704	0.055
Academic Motivation Scale								
Amotivation	1.96 (1.01)	1.90 (0.84)	0.683	0.064	1.91 (1.02)	2.04 (1.04)	0.264	0.126
External regulation	4.33 (0.74)	4.25 (0.94)	0.525	0.094	4.48 (0.61)	4.46 (0.65)	0.878	0.031
Introjected regulation	4.12 (0.82)	4.08 (0.94)	0.775	0.045	4.17 (0.74)	4.16 (0.77)	0.906	0.013
Identified regulation	4.13 (0.77)	4.02 (0.97)	0.433	0.125	4.27 (0.72)	4.18 (0.70)	0.412	0.126
IM-to know	4.09 (0.69)	3.94 (0.93)	0.199	0.183	4.21 (0.73)	3.99 (0.82)	0.046	0.283
IM-to accomplishment	4.21 (0.78)	4.10 (0.98)	0.387	0.124	4.29 (0.71)	4.14 (0.97)	0.151	0.176
IM-to stimulation experiences	3.57 (0.85)	3.48 (0.91)	0.518	0.102	3.81 (0.82)	3.69 (0.91)	0.397	0.138

Note. SEM: sports education model; TM: traditional methodology; PE: physical education; PEO: positive evaluation of others; IM: intrinsic motivation. Post- and pre-values are shown as mean (standard deviation).

**Table 5:** Per-protocol analysis shows the associations between changes in PE importance, prosocial improvement, and academic motivation after the 10-week intervention program for the intervention groups (TM and SEM).

Questionnaire			Model I		Model II	
	Changes in TM Group, Post–Pre (*n* = 64)	Changes in SEM Group, Post–Pre (*n* = 63)	Between-Group Difference ^a^ (95% Confidence Interval)	*p*-Value	Between-Group Difference ^a^ (95% Confidence Interval)	*p*-Value
PE Importance						
1. I think it is important to receive PE classes.	−0.343 (1.503)	0.079 (1.286)	0.264 (−0.034, 0.457)	0.091	0.264 (−0.377, 0.113)	0.101
2. I think that PE is one of the most important.	−0.156 (1.394)	0.222 (1.113)	0.066 (−0.033, 0.411)	0.094	0.066 (−0.181, 0.247)	0.106
3. I think the things I learn in PE will be useful in my life.	−0.453 (1.457)	0.095 (1.240)	0.358 (0.036, 0.512)	0.024	0.358 (−0.415, 0.057)	0.027
Prosocial Improvement						
Physical help	0.141 (1.531)	0.031 (1.575)	0.110 (−0.027, 0.379)	0.562	0.140 (−0.364, 0.224)	0.550
Physical service	−0.0140 (1.641)	0.015 (1.224)	0.125 (−0.176, 0.333)	0.544	0.125 (−0.318, 0.193)	0.545
Giving	0.046 (1.740)	0.507 (1.605)	0.461 (−0.064, 0.525)	0.123	0.465 (−0.018, 0.573)	0.122
Verbal help	−0.265 (1.300)	−0.095 (0.995)	0.170 (−0.118, 0.289)	0.409	0.163 (−0.384, 0.023)	0.431
Verbal comfort	−0.028 (1.463)	0.317 (1.044)	0.289 (0.076, 0.523)	0.009	0.036 (−0.206, 0.242)	0.010
PEO	−0.343 (1.287)	0.031 (1.217)	0.312 (−0.032, 0.408)	0.094	0.312 (−0.376, 0.064)	0.101
Deep listening	0.187 (1.435)	0.111 (0.935)	0.076 (−0.251, 0.175)	0.723	0.080 (−0.064, 0.363)	0.712
Empathy	0.265 (1.576)	0.317 (1.267)	0.233 (−0.225, 0.277)	0.839	0.061 (0.040, 0.543)	0.812
Solidarity	0.234 (1.306)	0.333 (1.257)	0.099 (−0.176, 0.275)	0.664	0.094 (0.058, 0.510)	0.680
Positive presence and unity	−0.078 (1.635)	0.063 (1.148)	0.015 (−0.178, 0.319)	0.574	0.015 (−0.245, 0.241)	0.606
Academic Motivation Scale						
Amotivation	−0.136 (0.970)	0.059 (1.152)	0.077 (−0.089, 0.285)	0.301	0.078 (−0.226, 0.149)	0.299
External regulation	0.015 (0.807)	0.075 (0.936)	0.060 (−0.124, 0.183)	0.701	0.061 (−0.109, 0.200)	0.699
Introjected regulation	0.011 (0.792)	0.039 (1.095)	0.028 (−0.154, 0.182)	0.869	0.021 (−0.142, 0.193)	0.900
Identified regulation	0.089 (0.869)	0.107 (1.077)	0.018 (−0.163, 0.180)	0.921	0.023 (−0.074, 0.271)	0.899
IM-to know	0.218 (0.859)	0.146 (0.897)	0.072 (−0.190, 0.118)	0.645	0.075 (0.028, 0.338)	0.632
IM-to accomplishment	0.152 (0.838)	0.111 (1.011)	0.041 (−0.184, 0.142)	0.803	0.056 (−0.280, 0.292)	0.729
IM-to stimulation experiences	0.109 (1.025)	0.091 (1.114)	0.018 (−0.197, 0.179)	0.924	0.025 (−0.088, 0.288)	0.895

Note. SEM: sports education model; TM: traditional methodology; PE: physical education; PEO: positive evaluation of others; IM: intrinsic motivation. Post- and pre-values are shown as mean (standard deviation). ^a^ Non-standardized indicates a difference between groups. The values are shown as the mean (standard deviation): Model I was unadjusted, and Model II was adjusted for gender (female/male/other option). The mean results show the differences between the post–pre-intervention results (i.e., between baseline and after a 10-week sport model program) for each variable, with negative values as a reduction in the post-evaluation compared with the baseline (standard deviation).

**Table 6:** Main adaptations made to SEM elements.

Element	Adaptations
Season	First unit for preseason and initial evaluation. Progressive autonomy for session design and practice. Competitions every three sessions until the final event.
Roles	The classic roles of the model are maintained, although their responsibilities vary depending on the nature of the activity. The role of the choreographer emerges. Other roles, such as doctor, nutritionist, and physical therapist, may be incorporated.
Affiliation	Membership at a gym is obtained, which must be named and identified with certain colors, a logo, and certain values.
Statistics	Results are recorded progressively and independently. Fitness test results are recorded online. Competition results are published in a virtual classroom (Moodle) or on a blog.
Final event	Two final events take place during the course. To provide them with greater significance, they are held as a complementary activity in locations other than the regular classroom.

## Data Availability

Datasets of this study are available on https://doi.org/10.6084/m9.figshare.28414793.

## Abbreviations

The following abbreviations are used in this manuscript:

HIIT	High Intensity Interval Training
AMRAP	As Many Rounds As Possible
IM	Intrinsic Motivation
CPAO	Confirmation and Positive Appraisal of the Other
U	Unit
S	Session
